# Leukocyte Telomere Length Independently Predicts 3-Year Diabetes Risk in a Longitudinal Study of Chinese Population

**DOI:** 10.1155/2020/9256107

**Published:** 2020-03-09

**Authors:** Yiwen Liu, Chifa Ma, Pingping Li, Chunxiao Ma, Shuli He, Fan Ping, Huabing Zhang, Wei Li, Lingling Xu, Yuxiu Li

**Affiliations:** ^1^Department of Endocrinology, Key Laboratory of Endocrinology, Ministry of Health, Peking Union Medical College Hospital, Peking Union Medical College, Chinese Academy of Medical Sciences, Beijing 100730, China; ^2^State Key Laboratory of Bioactive Substance and Function of Natural Medicines, Institute of Materia Medical Sciences and Peking Union Medical College, Beijing 100050, China; ^3^Diabetes Research Center of Chinese Academy of Medical Sciences, Beijing 100050, China; ^4^Department of Nutrition, Peking Union Medical College Hospital, Beijing 100730, China

## Abstract

Cellular aging markers, including telomere length and mitochondrial function, as well as oxidative stress and inflammation markers influence each other and form a complex network, which is affected in diabetes. However, it remains unknown whether these markers could independently predict future diabetes after adjustment for their mutual effects. We conducted a 3-year longitudinal study in a Chinese cohort that comprised 108 nondiabetic individuals at baseline. The 2-hour 75 g oral glucose tolerance tests were performed at baseline and at 3-year follow-up. At baseline, leukocyte telomere length (LTL) and mitochondrial DNA copy number (mtDNAcn) in leukocytes were determined using the polymerase chain reaction method. Tumor necrosis factor (TNF-*α*), interleukin-6, 8-hydroxy-2-deoxyguanosine levels, and superoxide dismutase (SOD) activity were measured by the enzyme-linked immunosorbent assay. Participants who developed diabetes at the 3-year follow-up (*n* = 28) had shorter LTL and higher levels of TNF-*α* and SOD activity at baseline. Baseline LTL was found to be independently associated with the development of diabetes at the 3-year follow-up after the adjustment for mtDNAcn, markers of oxidative stress and inflammation, and conventional diabetes risk factors. Our findings suggest that LTL is an independent predictor for 3-year diabetes risk, which might inform timely prevention and treatment of diabetes. Telomere shortening might be involved in the pathogenesis of diabetes independently of conventional diabetes risk factors, mtDNAcn, or oxidative stress and inflammation pathways.

## 1. Introduction

Over the last several decades, numerous studies have revealed that telomere length, mitochondrial function, oxidative stress, and inflammation are cellular aging markers intertwined in the complex relationships that determine their roles in cellular senescence [1–3]. These cellular aging markers have also been reported to be associated with various age-related diseases [4, 5], including diabetes [6, 7]. The relationships between these cellular aging markers and diabetes are particularly complex. The potential associations between leukocyte telomere length (LTL), mitochondrial DNA copy number (mtDNAcn), markers of oxidative stress/antioxidants, and proinflammatory cytokines on the one hand and diabetes on the other hand have been intensively scrutinized. Unfortunately, the cross-sectional design of the majority of the previous population-based studies failed to uncover causal relationships between these cellular aging markers and diabetes, with many findings being inconsistent [8–14]. Prospective studies are scarce, with only three published reports [15–17] investigating the prospective association of LTL with the development of diabetes. Furthermore, the results of those studies were discrepant and confusing. In a multiracial cohort of postmenopausal women, just a weak association was found between baseline LTL and future diabetes risk, which was attenuated after adjusting for conventional diabetes risk factors [15]. In contrast, in an American Indian cohort, baseline LTL was identified as a predictive marker independent of conventional diabetes risk factors [16]. Similar results were obtained in a European cohort [17]. The telomere length is characterized by strong heritability, and it varies substantially among different races [18], which could partly explain the discrepancy among these studies. Notably, none of these prospective studies included East Asian or, more specifically, Chinese subjects. Additionally, studies exploring the predictive role of mtDNAcn as well as markers of oxidative stress and inflammation in diabetes development are rare in general. Furthermore, considering the complex relationships between these biomarkers, it should be established whether they could affect each other's predictive role in diabetes risk. Taking into account these considerations, we conducted a 3-year longitudinal study in a Chinese population to investigate the prospective associations of baseline LTL and mtDNAcn, as well as oxidative stress and inflammatory markers with the development of diabetes 3 years later.

## 2. Methods

### 2.1. Study Participants

The current study was conducted in a Chinese cohort from the ChangPing suburb of Beijing between March 2014 and May 2017. The study protocols and consent procedures had been approved by the Ethics Committee of the Peking Union Medical College Hospital. All participants have signed the informed consent forms. A total of 142 individuals who had completed data in 2014 and 2017 were recruited into this study. According to the World Health Organization criteria [19], participants with fasting plasma glucose (FPG) < 7.0 mmol/L and 2-hour postload plasma glucose (PG) < 11.1 mmol/L during the 75 g oral glucose tolerance test (OGTT) were identified as nondiabetic individuals, including those with normal glucose tolerance (NGT) and prediabetes (impaired fasting glucose (6.1 mmol/L ≤ FPG < 7.0 mmol/L and 2hPG < 7.8 mmol/L) and/or impaired glucose tolerance (7.8 ≤ 2hPG < 11.1 mmol/L and FPG < 6.1 mmol/L)). There were 34 individuals who had diabetes at baseline and were thus excluded from the study. Hence, a total of 108 nondiabetic individuals at baseline were selected as the study population, including those with NGT (*n* = 55) and prediabetes (*n* = 53) at baseline. Demographic and anthropometric data were collected at baseline and at 3-year follow-up, including age, sex, height, weight, waist circumference (WC), and hip circumference (HC).

### 2.2. Biochemical Analysis

All the participants underwent a 75 g 2-hour OGTT, in which blood samples were drawn at four time points at baseline (0 min, 30 min, 60 min, and 120 min), whereas blood samples were obtained at two time points at the 3-year follow-up (0 min and 120 min). The levels of PG were determined by the glucose oxidase assay. Serum insulin (INS) and C-peptide (CP) were determined by the chemiluminescence immunoassay (Insulin IRI #02230141 (128434) and C-peptide #03649928 (129026), both from Siemens Medical Solutions Diagnostics, Tarrytown, NY, USA). Glycosylated hemoglobin (HbA1c) was measured by high-performance liquid chromatography, whereas total cholesterol (TC), total triglyceride (TG), high-density lipoprotein cholesterol (HDL-C), low-density lipoprotein cholesterol (LDL-C), uric acid (UA), creatinine (Cr), and urea were measured using an automatic analyzer.

### 2.3. Calculations of Insulin Sensitivity and *β*-Cell Function Indices

Based on the results of the 75 g 2-hour OGTT, we evaluated the sensitivity to insulin and *β*-cell function of the participants by using the following equations [20–22]:

(1) $$\begin{array}{c} \text{HOMA}‐\text{IR}=\frac{\text{fasting INS }\left(\text{mIU}/\text{L}\right)\times \text{FPG }\left(\text{mmol}/\text{L}\right)}{22.5},\\ \text{HOMA}‐\beta =\frac{20\times \text{fasting INS }\left(\text{mIU}/\text{L}\right)}{\text{FPG }\left(\text{mmol}/\text{L}\right)-3.5},\\ \text{Matsuda index}=\frac{1000}{\sqrt{\left(\text{FPG }\left(\text{mg}/\text{dL}\right)\times \text{fasting INS }\left(\mu \text{IU}/\text{mL}\right)\right)\times \left(\text{mean OGTT glucose concentration }\left(\text{mg}/\text{dL}\right)\times \text{mean OGTT insulin concentration }\left(\mu \text{IU}/\text{mL}\right)\right)}},\\ \text{Insulin secretion}‐\text{sensitivity index}-2\left(\text{ISSI}‐2\right)=\left(\frac{\text{Insulin AUC}}{\text{Glucose AUC}}\right)\times \text{Matsuda index}. \end{array}$$

### 2.4. LTL Assays

The LTL assays were performed based on the blood samples collected at baseline. The details of LTL measurement have been described in our previous publication [23]. LTL was determined as the relative ratio of the telomere repeat copy number to the single copy number (T/S ratio) using the monochrome multiplex quantitative polymerase chain reaction protocol [24].

### 2.5. Measurement of mtDNAcn in Peripheral Blood

mtDNAcn was measured based on the blood samples collected at baseline. As described previously [25], genomic DNA was extracted from leukocytes using a QIAamp DNA Blood Midi Kit (Qiagen, Hilden, Germany) and subsequently purified, diluted, and quantified using a NanoDrop 1000 Spectrophotometer (Thermo Fisher Scientific, Wilmington, DE, USA). The relative mtDNAcn value was determined using the real-time polymerase chain reaction and adjusted by simultaneously measuring the amount of nuclear DNA.

### 2.6. Assessment of Oxidative Stress and Inflammatory Markers

The measurements of oxidative stress and inflammatory markers were performed based on the blood samples collected at baseline. The measurements of oxidative stress and inflammatory markers have also been described in detail previously [23]. Superoxide dismutase (SOD) activity, as well as the levels of 8-hydroxy-2-deoxyguanosine (8-OHdG), tumor necrosis factor (TNF-*α*), and interleukine-6 (IL-6) were determined according to the manufacturer's instructions (Cloud-Clone Corp., Houston, USA). Absorbance kinetics were measured using an enzyme-linked immunosorbent assay reader.

### 2.7. Statistical Analysis

Statistical analysis was conducted using the SPSS 25.0 software package (IBM), R Studio (version 1.2.1335, https://www.r-project.org/), and GraphPad Prism 8.0 (https://www.graphpad.com). Continuous variables are presented as the mean ± standard error of the mean, whereas categorical variables are presented as percentages. To examine if the data were normally distributed, the Shapiro-Wilk and Kolmogorov-Smirnov tests were used. Normality transformations were performed on the variables that did not meet the normality assumptions using the appropriate formula when necessary. Comparisons of the continuous variables between groups were performed by Student's *t*-test or nonparametric Mann-Whitney's *U*-test, where appropriate. Pearson's or Spearman's correlation analysis was performed to determine the bivariate correlations, where appropriate. To explore whether mtDNAcn mediated the effect of TNF-*α* on 30-minute postload plasma glucose (PG30min), PROCESS macro Version 3.3 was used to generate simple mediation models with ordinary least squares. Mediation hypotheses were tested via a bias-corrected bootstrap method with 5,000 samples to calculate 95% confidence intervals (95% CI). If the 95% CI did not encompass zero, this represented statistical significance of the mediating effect. To identify the independent predictors for diabetes risk, multivariate logistic regression analysis was performed. In all comparisons, the statistical significance was set at *P* < 0.05.

## 3. Results

### 3.1. Relationships between LTL, TNF-*α* Level, and SOD Activity and the Risk of Diabetes Development

According to whether the participants developed diabetes at the 3-year follow-up, they were divided into progressors (*n* = 29) and nonprogressors (*n* = 79). Tables 1 and 2 present the differences in characteristics between progressors and nonprogressors at baseline and at the 3-year follow-up, respectively. The proportions of NGT subjects in progressors and nonprogressors at baseline were similar (51.7% versus 50.6%, *P* = 0.920). At baseline, the progressors had a significantly higher level of PG30min (*P* = 0.001) and PG60min (*P* = 0.01) than nonprogressors, with slightly higher HbA1c (*P* = 0.059). However, the differences of other conventional risk factors of diabetes at baseline, including age, BMI, WC, lipid profile, UA, insulin sensitivity indices, and insulin secretion indices between the two groups did not reach statistical significance (*P* > 0.05). Baseline age-adjusted LTL of progressors was significantly shorter than that of nonprogressors (*P* = 0.005). TNF-*α* levels (*P* = 0.009) and SOD activity (*P* = 0.031) of progressors were higher than those of nonprogressors, whereas we failed to observe statistically significant differences in the values of mtDNAcn (*P* = 0.221), IL-6 (*P* = 0.742), and 8-OHdG (*P* = 0.108) between groups. As expected, at the 3-year follow-up, HbA1c, FPG, PG120min, fasting insulin (FINS), CP120min, TG, and HOMA-IR values of progressors were significantly higher than those of nonprogressors.

### 3.2. Correlation of Cellular Aging Markers with Clinical Characteristics at Baseline

**Figure 1:**
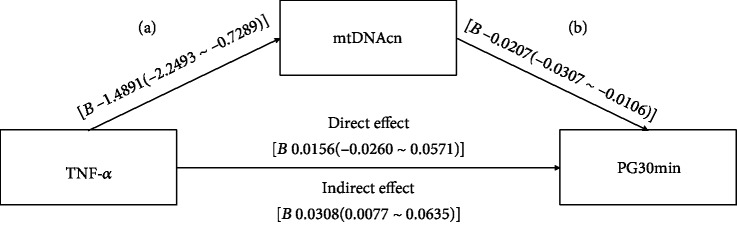
Mediation model of the association between TNF-*α*, mtDNAcn, and PG30min. The *P* values for regressions a and b are 0.0002 and 0.0001, respectively. The *P* value for the direct effect of TNF-*α* on PG30min is 0.4597. The absence of “0” in the 95% CI represents statistical significance of the indirect effect.

As indicated in Table 3, we found a modest positive correlation between baseline LTL and the Matsuda index (*r* = 0.208, *P* = 0.039), which proved to be a favorable OGTT-stimulated insulin sensitivity index. Nevertheless, we did not find an association between baseline LTL and any other conventional diabetes risk factor at baseline. mtDNAcn negatively correlated with PG30min, TG, and UA. The level of TNF-*α* positively correlated with HbA1c, PG30min, and UA, and negatively correlated with the Matsuda index. 8-OHdG level correlated with age, HDL-C, HOMA-IR, and HOMA-*β*. As for the correlations among the cellular aging markers per se, both LTL and mtDNAcn correlated with TNF-*α* level and SOD activity. To explore whether mtDNAcn mediated the effect of TNF-*α* on PG30min, mediation model analysis was performed. As shown in Figure 1, mtDNAcn fully mediated the effect of TNF-*α* on PG30min (direct effect *B* = 0.0156; 95% CI -0.0260–0.0571; indirect effect *B* = 0.0308; 95% CI 0.0077–0.0635).

### 3.3. Prospective Correlations of Baseline LTL, TNF-*α*, 8-OHdG, and SOD Activity with PG120min at the 3-Year Follow-Up

Subsequently, we performed Spearman's correlation analysis to explore the associations between parameters of cellular aging markers at baseline and clinical characteristics at the 3-year follow-up. As indicated in Table 4, baseline LTL negatively correlated whereas TNF-*α* level and SOD activity positively correlated with PG120min at the 3-year follow-up (*r* = −0.307, 0.324, and 0.272, respectively, *P* < 0.01). Baseline 8-OHdG level inversely correlated with PG120min and HOMA-*β* at the 3-year follow-up (*r* = −0.203, *P* < 0.05, and *r* = −0.257, *P* < 0.01, respectively). Additionally, both baseline LTL and TNF-*α* correlated with TC at the 3-year follow-up (*r* = −0.208 and 0.203, respectively, *P* < 0.05).

### 3.4. Independent and Prospective Association between LTL and Future Diabetes Risk

Multivariate logistic regression analysis was performed to explore the independent predictors for the 3-year diabetes risk. As shown in Table 5, age and sex-adjusted LTL at baseline were significantly associated with the development of diabetes at the 3-year follow-up (OR = 0.423, 95% CI 0.231–0.776, adjusted *P* = 0.005, Model 1). Following further and progressive adjustments for conventional diabetes risk factors (Model 2), as well as oxidative stress and inflammatory markers at baseline (Model 3), the association remained significant (Model 2: OR = 0.414, 95% CI 0.187–0.919, adjusted *P* = 0.030; Model 3: OR = 0.341, 95% CI 0.139–0.839, adjusted *P* = 0.019). As shown in [Supplementary-material supplementary-material-1], multivariate logistic regression analysis according to LTL quintiles demonstrated that individuals with LTL values in the second quintile were more than twelvefold likely to develop diabetes as those with the longest LTL values in the highest quintile (OR = 12.410, 95% CI 1.240–124.252, adjusted *P* = 0.032). However, the elevation of diabetes risk in individuals with LTL values in the first (OR = 8.626, 95% CI 0.646–115.257, adjusted *P* = 0.103), third (OR = 1.606, 95% CI 0.155–16.684, adjusted *P* = 0.691), and fourth quintiles (OR = 3.242, 95% CI 0.315–33.411, adjusted *P* = 0.323) compared to those in individuals with the longest LTL values in the highest quintile did not reach statistical significance, suggesting a nonlinear association between LTL at baseline and diabetes risk at the 3-year follow-up.

## 4. Discussion

Our longitudinal findings indicate that short LTL at baseline may be predictive of increased future diabetes risk. This relationship was independent of the effects of conventional diabetes risk factors, mtDNAcn, or oxidative stress and inflammatory markers. Therefore, telomere shortening is possibly involved in the pathogenesis of diabetes via the pathways other than those mediating the effects of conventional diabetes risk factors, mitochondrial dysfunction, oxidative stress, and inflammation.

Our observation that LTL predicts diabetes risk independently of conventional diabetes risk factors (e.g., age, BMI, WC, FPG, and lipid profile) was consistent with findings of previous prospective studies [16, 17]. We considerably extended the conclusions of those reports by showing that the correlation between LTL for diabetes risk is also independent of mtDNAcn and markers of oxidative stress and inflammation. Thus far, the role of the latter biomarkers in the development of diabetes remained confusing. Previous data indicated that telomere shortening and mitochondrial dysfunction likely affect each other and thus form a self-amplifying cycle [26]. The impaired mitochondria produce excessive reactive oxygen species that subsequently cause oxidative stress and inflammation [27]. In turn, oxidative stress and inflammation have also been demonstrated to damage telomere [28–30] and mtDNA [31, 32], which results in a vicious cycle. These changes may contribute to the development of diabetes via their possible involvement in insulin resistance [31, 33, 34] and *β*-cell dysfunction [35, 36]. Our finding of the positive correlation between LTL and the insulin sensitivity index, the Matsuda index, at baseline indicates an association between telomere shortening and resistance to insulin. The prospective inverse correlation between baseline 8-OHdG and HOMA-*β* at the 3-year follow-up suggests that oxidative DNA damage might participate in *β*-cell dysfunction, which is consistent with a previous finding of pancreatic *β*-cells being particularly vulnerable to oxidative damage [37]. The levels of 8-OHdG, a well-known biomarker of DNA oxidation [38], were demonstrated to be elevated in prediabetes and in patients with T2D in numerous previous retrospective studies [39, 40]. Unexpectedly, we found a modest inverse correlation between baseline 8-OHdG and PG120min at the 3-year follow-up, suggesting that elevated 8-OHdG might predict or contribute to decreased postload plasma glucose, which seemingly contradicts the previous findings. Nevertheless, considering the retrospective design of the previous studies and the lack of studies exploring the prospective association between 8-OHdG and future hyperglycemia, further large-scale prospective investigations are needed to validate our observations. Despite the important role of mitochondrial dysfunction, oxidative stress, and inflammation in the development of diabetes, the prospective association between LTL and the development of diabetes, which in our study was independent of mtDNAcn or markers of oxidative stress and inflammation, indicates that other potential pathways might mediate the effects of telomere shortening on the predisposition to diabetes. Further investigations are necessary to uncover mechanisms and pathways underlying this association.

To the best of our knowledge, this is the first study to explore an independent effect of mtDNAcn on the risk of future diabetes. We did not detect an independent effect of this parameter on the risk of diabetes at the 3-year follow-up. Another prospective study observed that adding mtDNAcn as an adjunctive marker augmented the predictive power of HbA1c and OGTT for future diabetes, but its independent role was not investigated [41]. Considering the small sample size of our present study, larger population studies are needed to validate our conclusion.

Several previous cross-sectional and prospective studies reported associations between LTL and the fasting insulin sensitivity index HOMA-IR [33, 42, 43]. In our correlation analysis, we found that LTL positively correlated with the Matsuda index at baseline, but not with HOMA-IR either at baseline or at the 3-year follow-up, which conflicts with the previous reports. Although the small sample size of the present study might partially explain this discrepancy, it is worth noting that these two indices provide different information: the Matsuda index reflects whole-body insulin sensitivity, whereas HOMA-IR has been recognized as an index of peripheral or hepatic insulin sensitivities based on the assumption that they are equivalent [21]. In addition, the Matsuda index has been reported to highly correlate with the gold standard whole-body insulin sensitivity index derived from euglycemic insulin clamp, suggesting that the Matsuda index provides a reasonable approximation of the whole-body insulin sensitivity [21]. Unfortunately, during the OGTT at the 3-year follow-up, PG30min and PG60min were not obtained; therefore, the value of the Matsuda index could not be calculated. The prospective association of LTL and the Matsuda index needs to be explored in future studies.

Interestingly, we found that both mtDNAcn and TNF-*α* correlated with PG30min. The mediation model analysis revealed that TNF-*α* indirectly contributed to the elevation of PG30min via the decrease of mtDNAcn, whereas its direct effect was not significant. Our findings indicated that TNF-*α* likely contributes to hyperglycemia predominately via mitochondrial dysfunction rather than other pathways, but this remains to be confirmed. Notably, except for PG30min, parameters such as FPG, PG60min, and PG120min did not correlate with mtDNAcn and TNF-*α*. After glucose loading, the first-phase insulin secretion occurs within 30 min, suppressing glucose production in the liver [44]. Impaired first-phase insulin secretion leads to the elevation of PG30min. Therefore, our result suggests that mtDNAcn and TNF-*α* are possibly associated with impaired first-phase insulin secretion.

Overall, we made several important observations in the present study. First, to the best of our knowledge, our analysis for the first time demonstrated that LTL independently predicted 3-year diabetes risk after adjusting for the confounding effects of mtDNAcn, oxidative stress, and inflammation marker levels, as well as of the conventional diabetes risk factors. Second, we investigated the associations between the cellular aging markers on the one hand and PG30min and PG60min values on the other hand, which were rarely determined in previous studies. Intriguingly, this is the first study that reports correlations of mtDNAcn and TNF-*α* level with PG30min, although the exact mechanisms underlying these relationships need to be clarified in future studies.

However, several limitations also need to be mentioned. The relatively small sample size limited statistical power and thereby likely precluded confirmation of the associations between some parameters that could be revealed in a larger cohort. This circumstance partially explains the absence of correlations among the indicators. On the other hand, the follow-up time was relatively short; thus, the earlier predictive value of these cellular aging markers was not explored in the current study.

## 5. Conclusion

Collectively, our observations suggest that LTL is an independent predictor for the 3-year diabetes risk. Its predictive role, which was independent of mtDNAcn and markers of oxidative stress and inflammation, as well as of conventional diabetes risk factors, might provide novel insights into the potential pathways that mediate the effect of telomere shortening on diabetes development. These specific pathways will need to be identified in future studies. Importantly, identification of telomere length as an additional risk modifier could assist early prediction for future diabetes and its timely prevention. However, large population-based studies are needed to validate the clinical value of our present findings.

## Figures and Tables

**Table 1:** Differences of baseline characteristics between progressors and nonprogressors.

Baseline characteristics	Progressors (*n* = 29)	Nonprogressors (*n* = 79)	*P* value
NGT (*n* (%))	15 (51.7%)	40 (50.6%)	0.920
Sex (male/female)	8/21	57/22	0.979
Age (years)	55.5 ± 1.8	54.1 ± 1.1	0.519
BMI (kg/m^2^)	26.12 ± 0.55	26.05 ± 0.47	0.930
WC (cm)	86.8 ± 2.0	86.4 ± 1.1	0.989
WHR	0.95 ± 0.00	0.95 ± 0.00	0.110
HbA1c (%)	5.8 ± 0.1	5.5 ± 0.1	0.059
FPG (mmol/L)	5.84 ± 0.12	5.85 ± 0.06	0.975
PG30min (mmol/L)	11.42 ± 0.43	9.69 ± 0.25	0.001
PG60min (mmol/L)	10.58 ± 0.84	9.14 ± 0.28	0.010
PG120min (mmol/L)	7.23 ± 0.42	6.89 ± 0.18	0.465
TC (mmol/L)	5.29 ± 0.24	5.48 ± 0.12	0.430
TG (mmol/L)	1.56 ± 0.15	1.60 ± 0.12	0.774
HDL-C (mmol/L)	1.29 ± 0.05	1.31 ± 0.03	0.734
LDL-C (mmol/L)	2.71 ± 0.17	2.84 ± 0.08	0.470
UA (*μ*mol/L)	274.2 ± 13.6	284.6 ± 9.4	0.605
Cr (*μ*mol/L)	73.1 ± 3.6	69.9 ± 2.2	0.465
Urea (*μ*mol/L)	5.15 ± 0.23	5.00 ± 0.14	0.606
HOMA-IR	2.97 ± 0.39	2.76 ± 0.20	0.796
Matsuda index	4.43 ± 0.69	4.86 ± 0.39	0.374
HOMA-*β*	99.77 ± 12.36	91.23 ± 5.90	0.813
ISSI-2	449.44 ± 37.81	517.01 ± 24.43	0.074
Age-adjusted LTL	28.21 ± 0.17	28.80 ± 0.11	0.005
mtDNAcn	96.69 ± 7.73	109.15 ± 5.44	0.221
TNF-*α* (fmol/mL)	29.88 ± 1.44	24.63 ± 1.31	0.009
IL-6 (pg/mL)	4.31 ± 0.63	4.23 ± 0.37	0.742
8-OHdG (pg/mL)	33.15 ± 3.83	43.14 ± 3.45	0.108
SOD (U/mL)	68.48 ± 2.96	62.36 ± 1.75	0.031

**Table 2:** Differences of clinical characteristics at the 3-year time point between progressors and nonprogressors.

Characteristics at 3-year time point	Progressors (*n* = 29)	Nonprogressors (*n* = 79)	*P* value
BMI (kg/m^2^)	26.34 ± 0.58	26.29 ± 0.47	0.901
WC (cm)	89.24 ± 1.53	87.92 ± 1.25	0.550
WHR	0.91 ± 0.02	0.87 ± 0.03	0.424
HbA1c (%)	5.59 ± 0.09	5.36 ± 0.04	0.011
FPG (mmol/L)	5.85 ± 0.16	5.38 ± 0.06	0.018
PG120min (mmol/L)	30.39 ± 4.45	7.12 ± 0.18	<0.001
FINS (mIU/L)	10.02 ± 1.15	7.63 ± 0.47	0.049
INS120min (mIU/L)	69.04 ± 12.56	43.30 ± 3.84	0.162
FCP (ng/mL)	1.74 ± 0.16	1.38 ± 0.06	0.075
CP120min (ng/mL)	8.37 ± 0.69	6.60 ± 0.35	0.027
TG (mmol/L)	1.99 ± 0.22	1.74 ± 0.17	0.111
TC (mmol/L)	4.89 ± 0.27	4.79 ± 0.10	0.738
HDL-C (mmol/L)	1.29 ± 0.05	1.23 ± 0.03	0.340
LDL-C (mmol/L)	3.05 ± 0.21	2.88 ± 0.09	0.464
Cr (*μ*mol/L)	65.48 ± 2.50	63.53 ± 1.47	0.644
UA (*μ*mol/L)	295.62 ± 15.94	297.26 ± 8.78	0.924
HOMA-IR	2.68 ± 0.34	1.75 ± 0.12	0.010
HOMA-*β*	89.23 ± 9.86	79.09 ± 5.32	0.392

Abbreviations: NGT—normal glucose tolerance; BMI—body mass index; HbA1c—glycosylated hemoglobin A1c; WC—waist circumference; WHR—waist hip ratio; FPG—fasting plasma glucose; PG—postload plasma glucose during OGTT; FINS—fasting insulin; FCP—fasting C-peptide; TC—total cholesterol; TG—total triglyceride; HDL-C—high-density lipoprotein cholesterol; LDL-C—low-density lipoprotein cholesterol; UA—uric acid; Cr—creatinine; LTL—leukocyte telomere length; mtDNAcn—mitochondrial DNA copy number; TNF-*α*—tumor necrosis factor-*α*; IL-6—interleukine-6; 8-OHdG—8-hydroxy-2-deoxyguanosine; SOD—superoxide dismutase; ISSI-2—insulin secretion-sensitivity index-2.

**Table 3:** Baseline correlates of clinical characteristics and cellular aging markers among the 108 participants.

Variables	LTL	mtDNAcn	TNF-*α*	Log2(IL-6)	8-OHdG	SOD
Sex	—	—^∗∗^	—	—	—	—
Age	0.007	-0.156	0.081	-0.053	0.230^∗^	0.193^∗^
BMI	-0.058	-0.126	0.114	-0.231^∗^	-0.111	-0.025
1/(WC)	-0.005	0.192	-0.140	0.164	0.042	-0.152
HbA1c	-0.169	-0.086	0.278^∗∗^	0.181	-0.012	0.234^∗^
FPG	-0.071	-0.124	-0.101	0.000	0.088	0.014
PG30min	-0.187	-0.420^∗∗^	0.200^∗^	0.027	0.030	0.130
PG60min	-0.160	-0.191	0.056	-0.030	-0.017	0.153
PG120min	-0.114	-0.057	0.088	0.258^∗∗^	0.014	0.034
NGT or prediabetes	—	—	—	—	—	—
TC	-0.047	-0.104	0.068	-0.048	0.174	-0.038
1/sqrt(TG)	0.069	0.223^∗^	-0.146	0.061	0.031	0.072
HDL-C	-0.131	-0.129	-0.007	0.100	0.197^∗^	0.079
LDL-C	-0.075	-0.083	0.112	-0.042	0.105	-0.074
UA	-0.196	-0.310^∗∗^	0.216^∗^	-0.005	-0.113	0.001
Log2(HOMA-IR)	-0.149	-0.057	0.155	-0.094	-0.204^∗^	-0.046
Lg(Matsuda index)	0.208^∗^	0.124	-0.197^∗^	0.079	0.174	0.013
Log2(HOMA-*β*)	-0.102	0.030	0.162	-0.125	-0.299^∗∗^	-0.088
1/sqrt(ISSI-2)	-0.133	-0.178	0.060	0.089	-0.076	0.091
LTL	1.000	0.080	-0.304^∗∗^	-0.195	-0.027	-0.268^∗∗^
mtDNAcn	0.080	1.000	-0.359^∗∗^	-0.022	-0.005	-0.213^∗^
TNF-*α*	-0.304^∗∗^	-0.359^∗∗^	1.000	0.125	-0.150	0.369^∗∗^
Log2(IL-6)	-0.195	-0.022	0.125	1.000	0.036	0.137
8-OHdG	-0.027	-0.005	-0.150	0.036	1.000	0.022
SOD activity	-0.268^∗∗^	-0.213^∗^	0.369^∗∗^	0.137	0.022	1.000

^∗^ *P* < 0.05. ^∗∗^*P* < 0.01. Abbreviations: BMI—body mass index; WC—waist circumference; HbA1c—glycosylated hemoglobin A1c; FPG—fasting plasma glucose; PG—plasma glucose after glucose load in OGTT; NGT—normal glucose tolerance; TC—total cholesterol; TG—total triglyceride; HDL-C—high-density lipoprotein cholesterol; LDL-C—low-density lipoprotein cholesterol; UA—uric acid; ISSI-2—insulin sensitivity index-2; LTL—leukocyte telomere length; mtDNAcn—mitochondrial DNA copy number; TNF-*α*—tumor necrosis factor-*α*; IL-6—interleukine-6; 8-OHdG—8-hydroxy-2-deoxyguanosine; SOD—superoxide dismutase.

**Table 4:** Spearman's correlation between baseline cellular aging markers and clinical characteristics at the 3-year follow-up.

Clinical characteristics at 3-year follow-up	Aging markers at baseline					
	LTL	mtDNAcn	TNF-*α*	IL-6	8-OHdG	SOD activity
HbA1c	-0.086	0.065	0.023	-0.098	0.092	-0.010
FPG	-0.008	-0.148	-0.108	-0.138	0.105	-0.073
PG120min	-0.307^∗∗^	-0.113	0.324^∗∗^	0.030	-0.203^∗^	0.272^∗∗^
FINS	-0.097	0.013	0.118	-0.164	-0.185	-0.125
INS120min	-0.133	-0.020	0.158	-0.120	-0.180	-0.075
FCP	-0.062	-0.037	0.121	-0.124	-0.206^∗^	-0.155
CP120min	-0.273^∗∗^	-0.126	0.251^∗^	-0.038	-0.227^∗^	0.019
HOMA-IR	-0.066	0.015	0.048	-0.118	-0.129	-0.097
HOMA-*β*	-0.090	0.080	0.123	-0.032	-0.257^∗∗^	-0.075
TC	-0.208^∗^	0.017	0.203^∗^	-0.031	0.109	0.020
TG	-0.084	-0.097	0.029	0.049	-0.076	-0.104
HDL-C	-0.079	-0.119	0.163	-0.039	0.104	0.197^∗^
LDL-C	-0.138	0.050	0.240^∗^	0.003	0.095	0.091
UA	-0.138	-0.181	0.114	-0.004	-0.182	-0.007

^∗^ *P* < 0.05. ^∗∗^*P* < 0.01. Abbreviations: HbA1c—glycosylated hemoglobin A1c; FPG—fasting plasma glucose; PG—plasma glucose; INS—insulin; CP—C-peptide; TG—total triglyceride; HDL-C—high-density lipoprotein cholesterol; LDL-C—low-density lipoprotein cholesterol; UA—uric acid; LTL—leukocyte telomere length; mtDNAcn—mitochondrial DNA copy number; TNF-*α*—tumor necrosis factor-*α*; IL-6—interleukine-6; 8-OHdG—8-hydroxy-2-deoxyguanosine; SOD—superoxide dismutase.

**Table 5:** Multivariate logistic regression models to evaluate the effect of covariates on the association between the baseline LTL and 3-year diabetes risk.

Models	Variables	Adjusted OR	95% CI	*P* values
Model 1	Age	1.010	0.962-1.060	0.699
	Male sex	1.080	0.389-2.996	0.883
	LTL	0.423	0.231-0.776	0.005

Model 2	Age	0.981	0.924-1.042	0.537
	Male sex	2.199	0.387-12.481	0.374
	BMI	0.999	0.801-1.247	0.993
	WC	1.009	0.937-1.087	0.807
	HbA1c	2.513	0.825-7.652	0.105
	FPG	0.555	0.175-1.765	0.319
	PG30min	1.517	0.984-2.339	0.059
	PG60min	1.157	0.779-1.718	0.470
	PG120min	0.869	0.568-1.330	0.518
	TG	0.691	0.325-1.469	0.337
	HDL-C	0.163	0.007-3.586	0.250
	LDL-C	0.898	0.328-2.455	0.833
	UA	0.994	0.985-1.004	0.239
	Matsuda index	1.050	0.852-1.293	0.648
	ISSI-2	0.756	0.174-3.274	0.708
	LTL	0.414	0.187-0.919	0.030

Model 3	Age	0.996	0.931-1.066	0.902
	Male sex	3.008	0.410-22.079	0.279
	BMI	0.944	0.746-1.196	0.636
	WC	1.003	0.931-1.081	0.939
	HbA1c	2.356	0.685-8.105	0.174
	FPG	0.608	0.169-2.184	0.445
	PG30min	1.501	0.930-2.422	0.097
	PG60min	1.094	0.705-1.697	0.689
	PG120min	1.004	0.628-1.604	0.988
	TG	0.656	0.283-1.520	0.326
	HDL-C	0.140	0.004-4.392	0.263
	LDL-C	0.904	0.305-2.675	0.855
	UA	0.994	0.984-1.003	0.191
	Matsuda index	1.129	0.902-1.413	0.289
	ISSI-2	0.635	0.143-2.817	0.550
	mtDNAcn	0.994	0.979-1.010	0.471
	TNF-*α*	1.007	0.933-1.086	0.865
	IL-6	0.874	0.721-1.059	0.169
	8-OHdG	0.977	0.950-1.005	0.108
	SOD activity	1.003	0.955-1.052	0.917
	LTL	0.341	0.139-0.839	0.019

Model 1: adjusted for age and sex. Model 2: adjusted for Model 1+conventional diabetes risk factors (BMI, WC, HbA1c, FPG, PG30min, PG60min, PG120min, TG, HDL-C, LDL-C, UA, Matsuda index, and ISSI-2). Model 3: adjusted for Model 2+mtDNAcn, as well as biomarkers related to oxidative stress and inflammation (mtDNAcn, TNF-*α*, IL-6, 8-OHdG, and SOD). Abbreviations: BMI—body mass index; WC—waist circumference; HbA1c—glycosylated hemoglobin A1c; FPG—fasting plasma glucose; PG—plasma glucose; TG—total triglyceride; HDL-C—high-density lipoprotein cholesterol; LDL-C—low-density lipoprotein cholesterol; UA—uric acid; ISSI-2—insulin sensitivity index-2; LTL—leukocyte telomere length; mtDNAcn—mitochondrial DNA copy number; TNF-*α*—tumor necrosis factor-*α*; IL-6—interleukine-6; 8-OHdG—8-hydroxy-2-deoxyguanosine; SOD—superoxide dismutase.

## Data Availability

The SPSS Statistics data used to support the findings of this study are available from the corresponding author upon request.

## Supplementary Materials

Supplementary Materials Table S1: multivariate-adjusted OR (95% CI) for diabetes risk and oxidative stress and inflammation according to baseline LTL quintiles.
