# Supplementary material for: Leukocyte Telomere Length Independently Predicts 3-Year Diabetes Risk in a Longitudinal Study of Chinese Population
Source: Oxid Med Cell Longev. 2020 Mar 9;2020:9256107. doi: 10.1155/2020/9256107 (PMC7085401; doi:10.1155/2020/9256107)
Supplement: Supplementary Materials — Table S1: multivariate-adjusted OR (95% CI) for diabetes risk and oxidative stress and inflammation according to baseline LTL quintiles. [file 9256107.f1.pdf]

Table S1. Multivariate-adjusted OR (95% CI) for diabetes risk and oxidative stress and inflammation according to baseline LTL quintiles.

LTL quintiles	OR	95%CI	Adjusted P-value
Q1 vs Q5	8.626	0.646-115.257	0.103
Q2 vs Q5	12.410	1.240-124.252	0.032
Q3 vs Q5	1.606	0.155-16.684	0.691
Q4 vs Q5	3.242	0.315-33.411	0.323

Adjusted for sex, age, BMI, WC, HbA1c, FPG, PG30min, PG60min, PG120min, TG, HDL-C, LDL-C, UA, Matsuda index, ISSI-2, mtDNAcn, TNF- α, IL-6, 8-OHdG, SOD activity

Abbreviations: Q, quintile; OR, odds ratios; 95%CI, 95% confidence interval; BMI, body mass index; WC, waist circumference; HbA1c, glycosylated hemoglobin A1c; FPG, fasting plasma glucose; PG, plasma glucose; TG, total triglyceride; HDL-C, high-density lipoprotein cholesterol; LDL-C, low-density lipoprotein cholesterol; UA, uric acid; ISSI-2, insulin-sensitivity index-2; LTL, leukocyte telomere length; mtDNAcn, mitochondrial DNA copy number; TNF-α,tumor necrosis factor-α； IL-6, interleukine-6; 8-OHdG, 8-hydroxy-2-deoxyguanosine; SOD, superoxide dismutase
